# Observation of coherent oscillation in single-passage Landau-Zener transitions

**DOI:** 10.1038/srep08463

**Published:** 2015-02-16

**Authors:** Guozhu Sun, Xueda Wen, Ming Gong, Dan-Wei Zhang, Yang Yu, Shi-Liang Zhu, Jian Chen, Peiheng Wu, Siyuan Han

**Affiliations:** 1 National Laboratory of Solid State Microstructures and Research Institute of Superconductor Electronics, School of Electronic Science and Engineering, Nanjing University, Nanjing 210093, China; 2 Synergetic Innovation Center of Quantum Information and Quantum Physics, University of Science and Technology of China, Hefei, Anhui 230026, China; 3 Department of Physics and Astronomy, University of Kansas, Lawrence, KS 66045, USA; 4 Department of Physics, University of Illinois at Urbana-Champaign, Urbana, IL 61801, USA; 5 National Laboratory of Solid State Microstructures, School of Physics, Nanjing University, Nanjing 210093, China

## Abstract

Landau-Zener transition (LZT) has been explored in a variety of physical systems for coherent population transfer between different quantum states. In recent years, there have been various proposals for applying LZT to quantum information processing because when compared to the methods using ac pulse for coherent population transfer, protocols based on LZT are less sensitive to timing errors. However, the effect of finite range of qubit energy available to LZT based state control operations has not been thoroughly examined. In this work, we show that using the well-known Landau-Zener formula in the vicinity of an avoided energy-level crossing will cause considerable errors due to coherent oscillation of the transition probability in a single-passage LZT experiment. The data agree well with the numerical simulations which take the transient dynamics of LZT into account. These results not only provide a closer view on the issue of finite-time LZT but also shed light on its effects on the quantum state manipulation.

Landau-Zener transition (LZT) has broad applications in atomic and molecular physics, quantum optics, condensed matter physics, chemical physics, and quantum information science. For example, LZT has been applied to investigating the jump time and quantum Zeno and anti-Zeno effects of cold atoms in accelerated optical lattices^1^,^2^, the behavior of molecular magnets at low temperature^3^,^4^, nonequilibrium phase transitions^5^, and it is also exploited as a tunable beam splitter of wave functions to generate entangled multipartite states^6^,^7^. LZT also plays a key role in determining whether random optimization problems can be solved using the quantum adiabatic algorithm^8^. Recently, LZT's potential for robust manipulation of coherent quantum states has attracted much attention in the context of quantum information processing^6^,^7^,^9^,^10^,^11^,^12^,^13^,^14^,^15^,^16^,^17^,^18^ because LZT may provide a simple and effective solution to the realization of high fidelity quantum state control without the need for precise timing.

The time-dependent Hamiltonian describing LZT in quantum two-level systems can be written in the generic form as
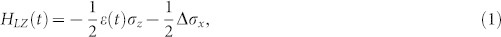
where *σ_x_*_,*z*_ are Pauli matrices, *ε*(*t*) = *vt* is the energy difference between the two diabatic (crossing) basis states (i.e., the eigenstates | ↑〉 and | ↓〉 of the *σ_z_* operator) controlled by an external parameter which depends linearly on time *t*, and Δ is the constant gap between the two instantaneous eigenenergy states |+〉 and |−〉 at the center of the avoided crossing *ε* = 0, as depicted in Fig. 1(a). In such systems, when *ε*(*t*) is swept through the avoided crossing, transitions between |±〉 with energies 
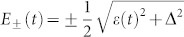
 can occur and the transition probability is given by the well-known Landau-Zener (LZ) formula
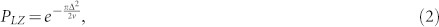
where *v* = |*dε*/*dt*| is the Landau-Zener speed and we have set the reduced Planck constant 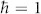. Equation (2) gives the probability of finding the system in the excited (ground) state at *ε_f_* = *ε*(*t* → +∞) when it is started in the ground (excited) state at *ε_i_* = *ε*(*t* → −∞). By defining *α* = Δ^2^/4*v* as the adiabaticity parameter the LZ formula can be simplified to *P_LZ_* = exp(−2*πα*). Although analytical solution to the problem cannot be obtained when *ε_i_* and/or *ε_f_* are finite, it is well known that for 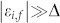, the LZ formula provides an excellent approximation to the actual transition probability and *P*_↓_ ≈ *P_LZ_*. However, when 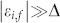 is not satisfied, the LZ formula may become quantitatively inaccurate or even qualitatively incorrect. In spite of some theoretical studies on the effects of finite |*ε_i_*_,*f*_|/Δ on *P*_↓_, there is an acute lack of adequate experimental evidence.

On the other hand, understanding LZT with moderate values of |*ε_i_*_,*f*_|/Δ is in urgent need because this region of parameter space is important to quantum information processing. For instance, in superconducting qubits the tuning range of energy level spacing is usually limited to a couple of GHz or even as narrow as a few hundreds of MHz while Δ/2*π* could be as large as 10^2^ MHz^6^,^7^,^19^,^20^,^21^,^22^,^23^,^24^,^25^,^26^. For quantum state control based on sweeping *ε* through avoided crossings, understanding LZT probability's dependence on |*ε_i_*_,*f*_|/Δ and the sweeping time is essential to high fidelity operation. The fidelity of various techniques based on LZT relies critically on the accuracy of the LZ formula which predicts a simple exponential dependence of *P_LZ_* on the adiabaticity parameter *α* only. Therefore, for a qubit starting from the ground (excited) state the probability of finding it in the excited (ground) state after a *single passage* through the avoided crossing is assumed to be determined entirely by *α* (i.e., Δ^2^/4*v*) but not the detail of the process such as |*ε_i_*_,*f*_|/Δ. Here, using an artificial atom — a superconducting phase qubit — coupled to a microscopic two-level system (TLS), we test the accuracy of the LZ formula in the region of |*ε_i_*_,*f*_|/Δ < 4.3. We show that in contrast to conventional wisdom, in the region of parameter space most relevant to superconducting qubits, *P*_↓_ could deviate significantly from *P_LZ_* determined by the LZ formula. Our experiment and numerical simulation demonstrate *P*_↓_ can oscillate coherently as a function of *ε_f_* for constant *α* when |*ε_i_*_,*f*_| is comparable to Δ, which is named as coherent Landau-Zener oscillation (LZO).

## Results

In our experiment we use a superconducting phase qubit. However, since a single phase qubit does not have an intrinsic avoided energy-level crossing, we utilize an avoided level crossing arising from interaction between the qubit and a microscopic TLS^27^,^28^. As discussed below in more detail, when the transition frequency of the qubit *ω*_10_ is close to that of the TLS *ω_TLS_*, which is fixed, the first and second excited states of the coupled qubit-TLS system form an effective quantum two-level system described by the LZ Hamiltonian (1). Note that to make quantitative comparisons between the theory/numerical simulation and the experiment without free parameters, all relevant system parameters, including the energy relaxation and dephasing time of the qubit and the energy gap Δ, are obtained from direct measurements.

**Figure 1:**
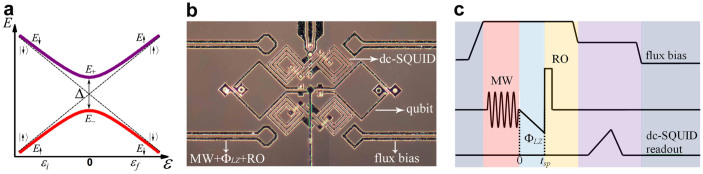
Circuit and experimental procedure. (a) A general avoided energy-level crossing with diabatic basis states (the dashed lines) and adiabatic basis states (the solid lines). The constant gap Δ between the two instantaneous eigenenergy states at *ε* = 0 is the tunneling amplitude, i.e., the coupling strength. (b) Optical micrograph of the sample with Al/AlO*_x_*/Al Josephson junctions on the silicon substrate. MW, Φ*_LZ_* and RO represent the microwave pulse to prepare the initial state, the sweeping flux bias to induce LZT and the 5-ns flux bias pulse to tilt the potential well in order to readout the qubit state, respectively. The planar coils and dc-SQUID magnetometer are coupled inductively to the qubit. (c) A typical time profile of the manipulation and measurement waveforms employed to perform the single-passage Landau-Zener experiment.

A microscopic picture of the superconducting phase qubit is shown in Fig. 1(b). The qubit consists of an *L* ≈ 770 pH superconducting loop intersected by a Al/AlO*_x_*/Al Josephson tunnel junction with a critical current *I*_0_ ≈ 1.4 *μ*A and a junction capacitance *C* ≈ 240 fF. By varying the magnetic flux applied to the superconducting loop the potential energy of the qubit becomes asymmetrical. The ground state and the first excited state in the upper potential well, represented by |0〉 and |1〉 respectively, can be used as the computational basis states of the qubit. For an isolated qubit, the transition frequency between |0〉 and |1〉, *ω*_10_, is a single-valued function of the external flux bias Φ*_x_* which is coupled inductively to the superconducting loop through an on-chip flux bias line.

**Figure 2:**
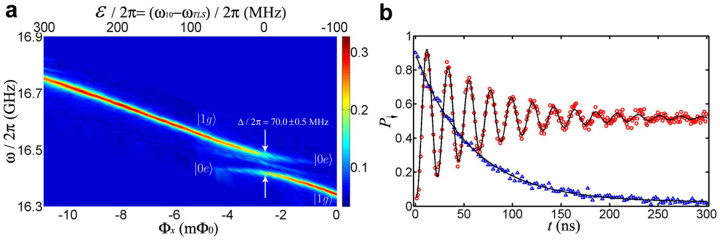
Spectroscopy and Rabi oscillation. (a) Microwave spectroscopy measurement of the coupled qubit-TLS system. The splitting is Δ/2*π* = 70.0 ± 0.5 MHz centered at *ω*/2*π* = 16.450 ± 0.002 GHz. The beginning point of the flux bias for the single passage LZ sweeping is denoted as 0 mΦ_0_, corresponding to *ε_i_*/2*π* = (*ω*_10_ − *ω_TLS_*)/2*π* ≈ −100 MHz in the upper abscissa. (b) Rabi oscillation and *T*_1_ at *ε_i_*, respectively. The experimental data (the red circles and blue triangles) agree well with the theoretical fits (the black solid lines).

As shown in Fig. 2(a), however, the microwave spectrum of the qubit *ω*_10_(Φ*_x_*) has a rather large avoided energy-level crossing at Φ*_x_* ≈ −2.8 mΦ_0_ (with respect to the flux bias point at which *ω*_10_/2*π* ≈ 16.348 GHz) indicating significant interaction between the qubit and a microscopic TLS^30^. The transition frequency between the TLS' ground state |*g*〉 and excited state |*e*〉 and the qubit-TLS coupling strength are *ω_TLS_*/2*π* = 16.450 ± 0.002 GHz and Δ/2*π* = 70.0 ± 0.5 MHz from the spectrum and vacuum Rabi oscillation, respectively. Note that in this coupled qubit-TLS system the time-dependent energy difference between the two diabatic states involved in LZT is *ε*(*t*) = *ω*_10_(*t*) − *ω_TLS_* which depends linearly on the flux bias to a good approximation. The relationship between the flux bias and *ε* can be found from Fig. 2(a).

Fig. 1(c) illustrates the experimental procedure used to observe coherent LZO. We begin by setting the initial diabatic energy of the effective quantum two-level system *ε_i_* at about 100 MHz below *ω_TLS_* with a static flux bias. The qubit is prepared in its ground state by waiting for much longer than the energy relaxation time *T*_1_ ≈ 70 ns of the qubit. A microwave pulse is then applied to the qubit when it is biased at a fixed value *ε_i_*/2*π* ≈ −100 MHz. The microwave pulse coherently transfers the population of the qubit-TLS from |0*g*〉 to one of the system's eigenstates |−〉 through a process that is discussed in detail in Methods. The lack of oscillation in *T*_1_ measurement taken at *ε_i_* as shown in Fig. 2(b) confirms that the initial state of the qubit-TLS system at *t* = 0 is indeed the eigenstate |−〉. As illustrated in Fig. 1(c), a time-dependent flux Φ(*t*) = Φ*_LZ_t*/*t_sp_* is then superimposed between *t* = 0 and *t* = *t_sp_* onto the static flux bias to sweep *ε* linearly from ~ −100 MHz to its maximum value *ε_f_*. The corresponding LZ speed *v* is thus (*ε_f_* − *ε_i_*)/*t_sp_*. This is followed immediately by a 5-ns readout pulse which performs a projective measurement of the probability *P*_↓_ of finding the qubit in state |1〉 (i.e., the coupled system is in state |1*g*〉 corresponding to | ↓〉 in Fig. 1(a)).

**Figure 3:**
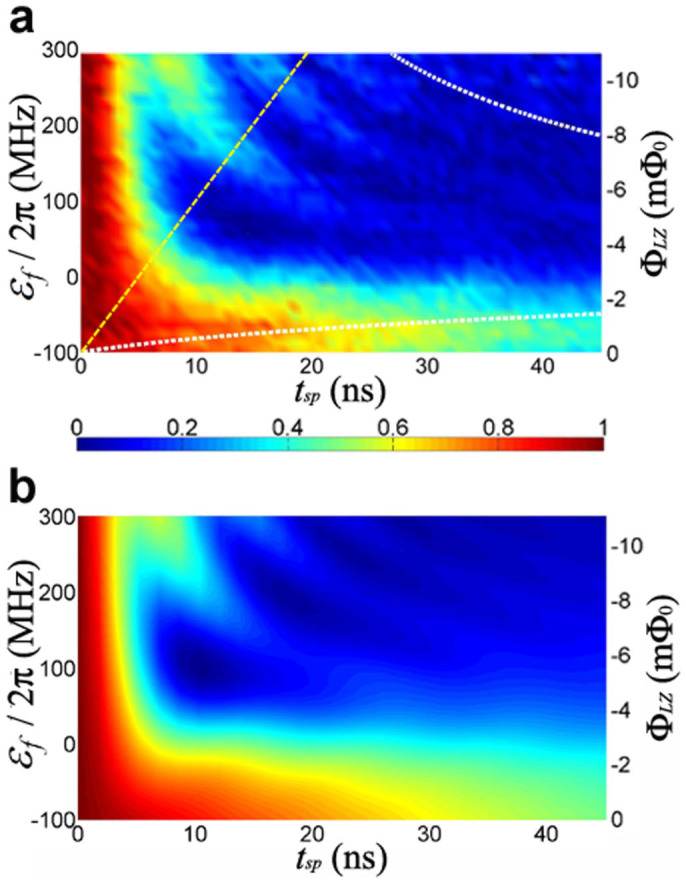
Coherent LZO. (a) Experimentally measured *P*_↓_ vs. *ε_f_* and *t_sp_*. (b) Numerically calculated *P*_↓_ vs. *ε_f_* and *t_sp_* with all input parameters obtained from the experiment. The white dashed lines correspond to the value of modified adiabaticity parameter *α*′ = 10. Notice that in the region below (above) the lower (upper) white dashed line, one has *α*′ ≫ 1 thus the system evolves adiabatically and no oscillation in *P*_↓_ is expected as confirmed experimentally. The LZ speed *v* equals the slope of any straight lines originated from the lower-left corner of the *t_sp_*-*ε_f_* plane. For example, the yellow dashed line in (a) has *v* = 400/19.5 ≈ 20.5 MHz/ns. For the sake of clarity, the temporal evolution of the system along the yellow dashed line (constant *v*) is presented separately in Fig. 4(b).

We first measure *P*_↓_ vs. *t_sp_* at a constant value of *ε_f_* by keeping Φ*_LZ_* fixed while increasing *t_sp_* from almost 0 ns to 45 ns. The maximum *t_sp_* is selected to avoid too much influence of the qubit's energy relaxation. By stepping Φ*_LZ_* from 0 to −11 mΦ_0_ the value of corresponding *ε_f_* is then varied from about −1.4Δ to ~ 4Δ, in which the condition 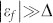 is no longer satisfied. This procedure is repeated at each *ε_f_* to obtain *P*_↓_(*ε_f_*, *t_sp_*). Fig. 3(a) shows the dependence of *P*_↓_ on *ε_f_* and *t_sp_*. It can be seen that *P*_↓_ vs. *t_sp_* decays exponentially for *ε_f_* < 0 (Φ*_LZ_* ∈ [−2.8 mΦ_0_, 0]) with a characteristic time *T*_1_ due to energy relaxation. As *ε_f_* becomes positive, *P*_↓_ vs. *t_sp_* becomes oscillatory. Since the avoided crossing is traversed only once, the observed oscillation in *P*_↓_ vs. *t_sp_* with constant *ε_f_* must be a consequence of the moderate value of *ε_f_*/Δ and is not caused by the Landau-Zener-Stückelberg interference which requires multiple passages through the avoided crossing.

It is worth noting that the observed oscillation is not a consequence of ill-prepared initial states with non-negligible probability amplitude in the excited state |+〉 of the effective Hamiltonian (1) because the microwave pulse used to initialize the system resonantly couples |0*g*〉 to |−〉 and has negligible coupling to |+〉 due to large frequency detuning. Furthermore, this process of transferring the system to the desired initial state |−〉 via a resonant microwave pulse is robust in the sense that it does not depend sensitively on the accuracy of the pulse duration *t_MW_*. Deviation in pulse duration simply leaves some probability amplitude in |0*g*〉 which has no effect on LZT other than reducing the visibility of the oscillation (see Methods for detail on the initial state preparation). Therefore, we are confident that the oscillation observed in the non-adiabatic region of the parameter space arises neither from Landau-Zener-Stückelberg interference nor unwanted probability amplitude of |+〉 in the initial state. This is also supported by the good agreement between the results of experiment and numerical simulation shown in Fig. 3(b), which uses |−〉 as the initial state at the start of the single passage sweep.

## Discussion

By replacing *ε_f_* with *ε_i_* + *vt*, solving the problem of sweeping *ε* in a finite range is transformed to finding *P*_↓_ at finite time. Previous studies have discovered that LZ transition probability reaches the asymptotic value given by equation (2) at 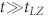 (assume *ε* = 0 at *t* = 0), where 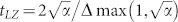 is called the Landau-Zener time, and oscillates in the vicinity of avoided crossing (corresponds to *t* ≤ *t_LZ_*) due to the transient dynamics^12^,^31^,^32^. Since corrections to the standard LZ formula are significant only if the adiabaticity parameter *α* ≤ 1, the region of *ε* within which transient dynamics plays an important role is given by 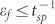, where “≤” means less than or comparable to. By examining the experimental data we find that the region of most noticeable coherent LZO coincides with 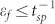 which agrees well with the result of numerical simulation. These results unambiguously show that coherent LZO is originated from the transient dynamics of the LZT.

Such coherent LZO has little effect on the adiabatic evolution. Because in the true adiabatic regime, by definition the system always stays in the instantaneous ground state and no LZT could occur. In order to find the region of approximate adiabatic evolution in our experiment, it is necessary to modify the definition of the adiabaticity parameter to 
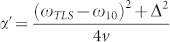
. For the adiabatic theorem to hold 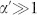 is required. As shown in Fig. 3(a), the white dashed lines represent *α*′ = 10. It is clear that there is no coherent LZO observed in the region 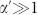.

**Figure 4:**
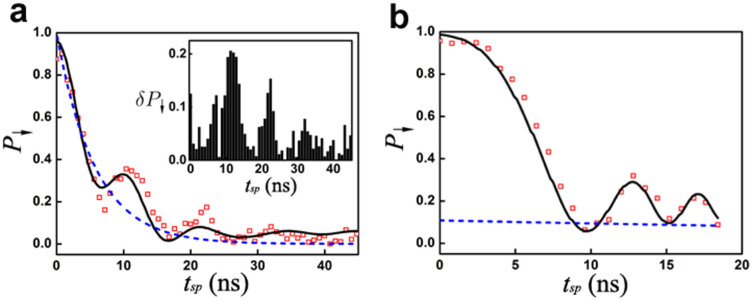
*P*_↓_ oscillation at constant LZ speed. (a) Measured *P*_↓_ as a function of *t_sp_* (the red squares) with *ε_f_*/2*π* = 200 MHz which clearly shows oscillation in the region of *α* < 1 which compares well with the result of numerical calculation (the solid line). This is in stark contrast to the smooth exponential decay expected from the LZ formula (the dashed line). The inset is the difference *δP*_↓_ = *P*_↓_ − *P_LZ_*. (b) The measured (the red squares) and numerically calculated (the solid line) *P*_↓_ vs. *t_sp_* with constant LZ speed *v* ≈ 20.5 MHz/ns corresponding to evolving along the yellow dashed line in Fig. 3(a). Again, *P*_↓_ oscillates in the region where the adiabatic condition *α* > 1 is not satisfied which is not expected from the LZ formula (the dashed line).

In a popular analogy to optics an avoided crossing acts as an effective beam splitter, with a transmission coefficient corresponding to *P_LZ_* in the LZ formula, for quantum wave functions. This beam splitter analogy has been applied successfully to the visualization and explanation of the behavior of superconducting and semiconductor qubits^6^,^9^,^19^,^20^,^33^,^34^,^35^,^36^. In this analogy, a single sweep through the avoided crossing is equivalent to passing a beam of light through the beam splitter only once. When 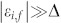, *P*_↓_ ≈ *P_LZ_* and thus a greater LZ speed corresponds to a higher transmission coefficient of the beam splitter according to the LZ formula. But when 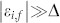 is not satisfied, *P*_↓_ differs greatly from *P_LZ_*. As an example, *P*_↓_ vs. *t_sp_*, and thus the LZ speed *v*, with *ε_f_*/2*π* = 200 MHz is shown in Fig. 4(a). The maximum in the difference *δP*_↓_ between the experimental *P*_↓_ and those obtained from the LZ formula (2), shown in the inset of Fig. 4(a), can reach 0.21. The observation of coherent LZO strongly suggests that when 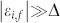 is not met corrections to the LZ formula should be considered to avoid conceptual difficulties.

Coherent LZO also has significant consequences on the coherent manipulation of quantum states of single qubits and coupled two-qubit systems based on LZT^10^,^12^. For this approach of quantum state control, the LZ transition probability *P_LZ_* plays a central role since each single passage through the avoided crossing results in a unitary operation *U_LZ_* given by Ref. 12
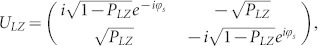
where *φ_s_* is the Stokes phase^37^, which has no effect on the single-passage LZT process discussed here and thus can be set to zero for the sake of convenience. As mentioned above, the transition frequency *ω*_10_ of most artificial atoms, in particular the superconducting qubits, is limited to a couple of GHz. Because the speed of two-qubit operations is proportional to the inter-qubit coupling strength Δ, increasing |*ε_i_*_,*f*_|/Δ by reducing Δ is undesirable. Hence, evaluating *U_LZ_* according to the LZ formula (2) could result in significant errors when 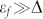 is not satisfied. In order to conduct a quantitative analysis, the experimental data along the yellow dashed line corresponding to constant *v*, in the Fig. 3(a) are extracted and shown in Fig. 4(b). Oscillation in *P*_↓_ is clearly observed and it is qualitatively different from the exponential decay predicted by equation (2), when decoherence is taken into account. Suppose the initial quantum state is | ↓〉. Then after a single passage through the avoided crossing, if one replaces *P_LZ_* with *P*_↓_ as in the asymptotic situation, the deviation *δP*_↓_ = *P*_↓_ − *P_LZ_* would be quite large. For example, when *t_sp_* = 12.8 ns the deviation *δP*_↓_ = 0.229, which is unacceptably large for coherent quantum state transformation.

In conclusion, we have investigated the effect of finite energy (*ε*) sweep (or equivalently finite time) on LZT probability *P*_↓_ experimentally. Single-passage technique is used to isolate the effect of finite *ε_f_* on *P*_↓_ from that of interference caused by passing the avoided crossing multiple times. We find that *P*_↓_(*ε_f_*/Δ, *α* = *const*) oscillates when *ε_f_* is comparable to Δ and *α* < 1. The good agreement between the experiment and numerical calculation strongly supports the notion that coherent LZO is caused by the underlying transient dynamics of the finite time LZT which cannot be described by the LZ asymptotic formula. In this region of the LZT parameter space, corrections to the LZ formula must be taken into account, otherwise it will lead to substantial errors in quantum state operations based on LZT. The result also shows that when applying the simple beam splitter analogy one should not automatically assume that greater *α* (i.e., faster sweep) corresponds to larger transmission coefficient (i.e., greater *P_LZ_*) as implied by the asymptotic LZ formula.

## Methods

### Initial state preparation

We first derive an analytical result explaining the lack of oscillation at the very beginning of LZT. The Hamiltonian of the qubit-TLS system coupled to a microwave field is given as: (in the basis {|0*g*〉, |1*g*〉, |0*e*〉, |1*e*〉})
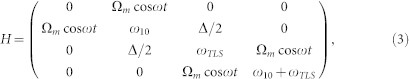
where Ω*_m_* is the Rabi frequency, *ω* is the microwave frequency, *ω_TLS_* is the energy difference between the ground state |*g*〉 and the excited state |*e*〉 of TLS, Δ is the coupling strength between the qubit and TLS, *δ* = *ω*_10_ − *ω* and *δ_r_* = *ω_TLS_* − *ω*_10_ are detunings. By rotating the frame, Hamiltonian (3) can be transformed to the following time-independent form^29^,^30^
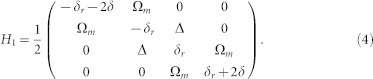
Next, we rewrite *H*_1_ in which the subspace spanned by {|1*g*〉, |0*e*〉} is diagonalized:
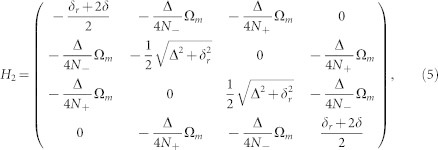
where 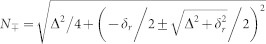. The basis for *H*_2_ is now {|0*g*〉, |−〉, |+〉, |1*e*〉}. Note that in our experiment, before turning on the microwave the state is at Ψ(*t* = 0) = |0*g*〉. By turning on the microwave (Ω*_m_* ≠ 0), |0*g*〉 is coupled to both |−〉 and |+〉. The resonance between |0*g*〉 and |±〉 occurs when 
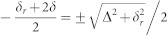
, from which we obtain the resonant condition:
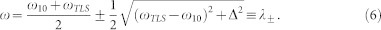
In the limit of 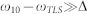, we have *ω* = *ω*_10_, which corresponds to the usual two-state Rabi oscillation. Note that in this limit there is also a solution *ω* = *ω_TLS_*. However, in this case the coupling strength is 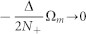. The reason is that although the microwave frequency could match that of TLS, coupling between the microwave and TLS is negligible which is confirmed by the absence of Rabi oscillation between the two states of the TLS in a separate experiment. In the other limit of *ω*_10_ − *ω_TLS_* = 0, we have 
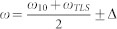
, and the dynamics have been thoroughly studied in Ref. 30.

In our experiment, we have (*ω_TLS_* − *ω*_10_)/2*π* ≈ 100 MHz and Δ/2*π* ≈ 70 MHz, which means LZT occurs in the region where (*ω_TLS_* − *ω*_10_) ~ Δ. Because the frequency of the applied microwave is *ω* = *λ*_−_, which can be determined from the measured energy spectrum shown in Fig. 2(a), |0*g*〉 is resonantly coupled to |−〉, which is the eigenstate of 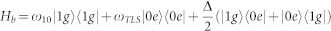. Although there is in principle also a coupling between |0*g*〉 and |+〉, the effective coupling is much smaller because of the large detuning, as discussed below.

For Δ/2*π* ≈ 70 MHz, (*ω_TLS_* − *ω*_10_)/2*π* ≈ 100 MHz, the resonance between |0*g*〉 and |−〉 occurs at 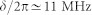, we obtain 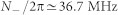 and 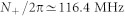. In our experiments, the coupling strength between |0*g*〉 and |−〉 is about 20 MHz and that between |0*g*〉 and |+〉 would be 
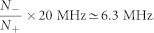
. Because 6.3 MHz is comparable with 20 MHz, one may think that coupling between |0*g*〉 and |+〉 cannot be neglected. However, there is also a large detuning of about 122 MHz between |0*g*〉 and |+〉. Therefore, the effective coupling between |0*g*〉 and |+〉 is reduced to (6.3^2^/122) ≈ 0.33 MHz and thus can be safely neglected. To be more precise, we calculated the population *P*_±_ (where *P*_±_ is the population of state |±〉) after the application of a *π* pulse numerically, and it is found that 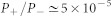. Based on this analysis, when *ω* = *λ*_−_, the dynamics can be described by the Hamiltonian in the subspace {|0*g*〉, |−〉}:
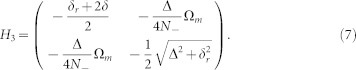
At the resonance 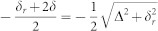, *H*_3_ becomes
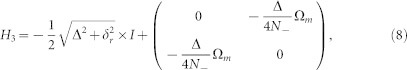
where *I* is a 2 × 2 identity matrix.

For initial state Ψ(*t* = 0) = |0*g*〉, the amplitude of |−〉 is
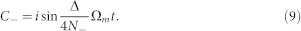
Considering 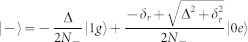, the amplitude of |1〉 is
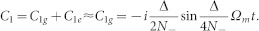


Therefore, with a microwave pulse of duration *t_MW_* which is used to prepare the initial state, we have
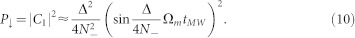
This is the reason why in the experiment we observe a usual Rabi oscillation instead of Rabi beating^30^ which is indicated by the red circles, as shown in Fig. 2(b). In addition, when microwave is turned off, the subspace {|+〉, |−〉} is isolated from |0*g*〉 and |1*e*〉. Projected into the subspace {|+〉, |−〉}, the system stays in the eigenstate |−〉. This explains why we observe a monotone decay of *P*_↓_ with no oscillations, as indicated by the blue triangles in Fig. 2(b).

### Effect of *t_MW_* and *ε_f_*/Δ on LZT

In this section, we discuss two factors that may affect the LZT probability, i.e., the width of the microwave pulse *t_MW_* used to prepare the initial state at *ε_i_* and the end of the normalized diabatic energy sweeping *ε_f_*/Δ, respectively.

**Figure 5:**
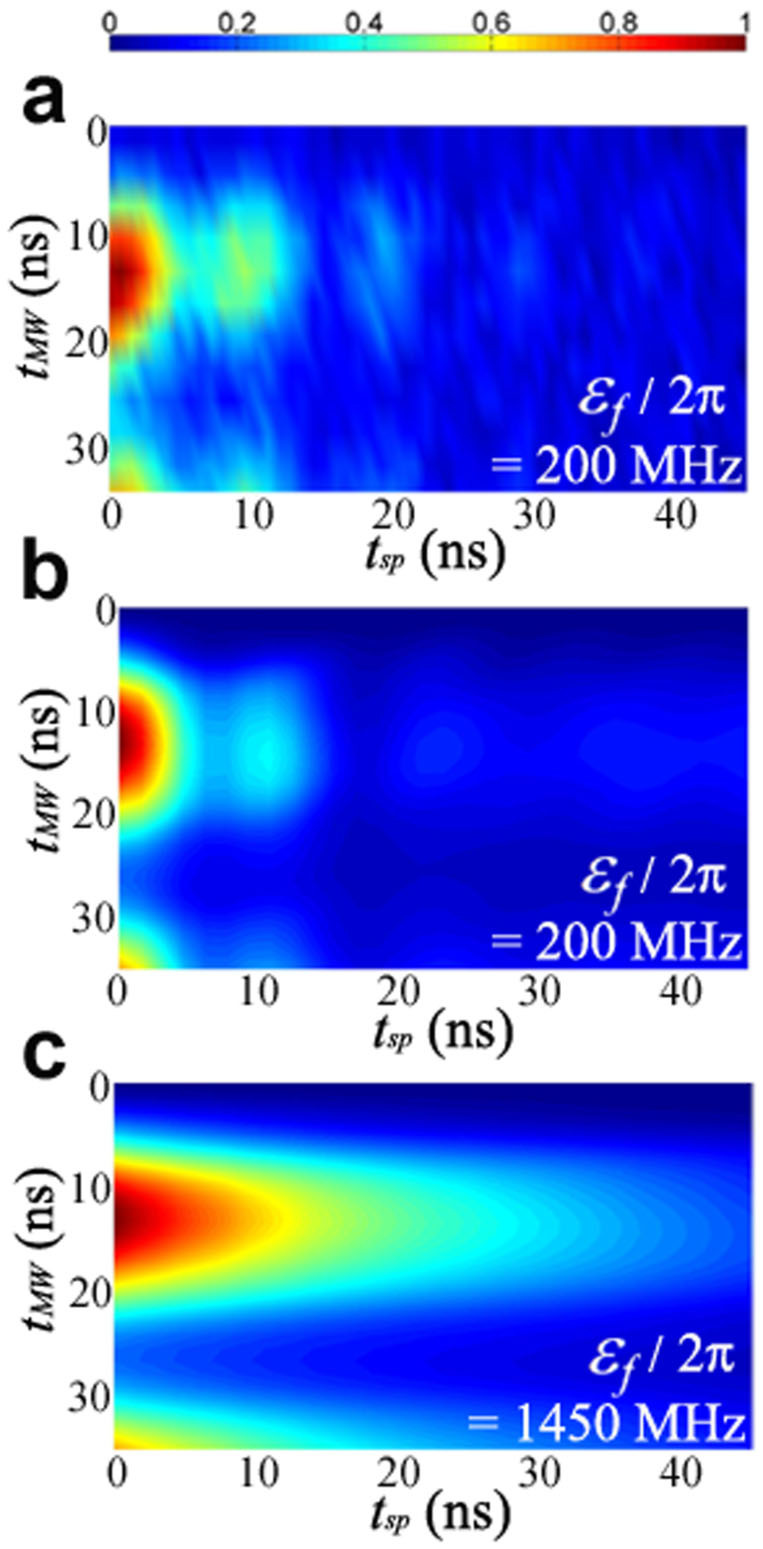
The effect of *t_MW_* on finite time LZT (a) Experimentally measured and (b) numerically calculated *P*_↓_ vs. *t_MW_* and *t_sp_* for *ε_f_*/2*π* = 200 MHz showing that the effect of imprecise *π* pulse is to reduce the visibility of the oscillation of *P*_↓_ vs. *t_sp_* by reducing the probability amplitude of the desired |−〉 state. Because the microwave pulse is resonant with the |0*g*〉 ↔ |−〉 transition while largely detuned from the |0*g*〉 ↔ |+〉 transition even a significant deviation from a *π* pulse would only result in negligible transfer of population to |+〉. Furthermore, since |0*g*〉 does not participate in the single passage LZ process, the observed oscillation could neither be due to LZS interference nor non-negligible population in |+〉 at the beginning of each *ε* sweep. For comparison, we also present the numerically calculated *P*_↓_(*t_MW_*, *t_sp_*) for *ε_f_*/2*π* = 1450 ≈ 20.7Δ in (c). The result shows the exponential decay behavior described by the asymptotic LZ formula as expected for 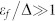.

After a microwave pulse, by projecting into the subspace {|1*g*〉, |0*e*〉}, the system is in the eigenstate |−〉. Then the dynamics of LZT can be described by *H_b_* with a time-dependent *ω*_10_(*t*), i.e.,
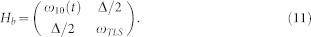
To investigate the Landau-Zener diffraction effect, we sweep *ω*_10_(*t*) across *ω_TLS_*, i.e.,
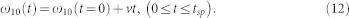
When 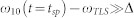, we expect that the Landau-Zener asymptotic formula holds and *P*_↓_ ≈ *P_LZ_*. Thus no oscillations should occur in *P*_↓_. This is confirmed by the result of numerical simulation shown in Fig. 5(c), where *ε_f_*/2*π* = 1450 MHz ≈ 20.7Δ, reproducing the exponential decay behavior described by the asymptotic LZ formula independent of the initial state of the qubit-TLS system. In this case, the population in the qubit state |1〉 can be expressed as
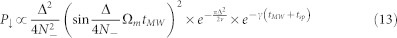
where the first term reflects the effect of microwave duration *t_MW_* in preparing the initial state, the second term corresponds to the LZT probability, and the third term represents the relaxation effect. However, as *ω*_10_(*t* = *t_sp_*) moves towards *ω_TLS_*, the situation 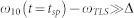 does not hold any more, and we observe oscillation features in the *t_sp_* direction, as shown in Fig. 5(a) (experiment) and 5(b) (numerical simulation). Notice that Fig. 5(a) and 5(b) also confirm that the effect of imprecise *π* pulse is an incomplete transfer of system from |0*g*〉 to |−〉, which reduces the probability amplitude of |−〉 from the maximum value, instead of resulting in non-negligible probability amplitude in the unwanted |+〉 state.

## Author Contributions

G.S. and S.H. conceived the experiments; G.S. carried out the measurements and analyzed the data with the help of X.W., M.G., D.Z., Y.Y., S.Z., J.C., P.W. and S.H.; X.W. performed the numerical calculations; G.S. and S.H. wrote the paper.

## References

[b1] Fischer M. C., Gutiérrez-Medina B. & Raizen M. G. Observation of the quantum zeno and anti-zeno effects in an unstable system. Phys. Rev. Lett. 87, 040402 (2001). 11461604 10.1103/PhysRevLett.87.040402

[b2] Zenesini A. *et al.* Time-resolved measurement of landau-zener tunneling in periodic potentials. Phys. Rev. Lett. 103, 090403 (2009). 10.1103/physrevlett.103.090403 19792769

[b3] Wernsdorfer W. & Sessoli R. Quantum phase interference and parity effects in magnetic molecular clusters. Science 284, 133–135 (1999). 10102810 10.1126/science.284.5411.133

[b4] Vijayaraghavan A. & Garg A. Incoherent landau-zener-stuckelberg transitions in single-molecule magnets. Phys. Rev. B 79, 104423 (2009).

[b5] Damski B. The simplest quantum model supporting the kibble-zurek mechanism of topological defect production: Landau-zener transitions from a new perspective. Phys. Rev. Lett. 95, 035701 (2005). 16090756 10.1103/PhysRevLett.95.035701

[b6] Sun G. *et al.* Tunable quantum beam splitters for coherent manipulation of a solid-state tripartite qubit system. Nat. Commun. 1, 51 (2010). 20975719 10.1038/ncomms1050 PMC2982164

[b7] Quintana C. M. *et al.* Cavity-mediated entanglement generation via landau-zener interferometry. Phys. Rev. Lett. 110, 173603 (2013). 23679727 10.1103/PhysRevLett.110.173603

[b8] Bapst V., Foini L., Krzakala F., Semerjian G. & Zamponi F. The quantum adiabatic algorithm applied to random optimization problems: The quantum spin glass perspective. Phys. Rep. 523, 127–205 (2013).

[b9] Zurek W. H. Cosmological experiments in superfluid helium? Nature 317, 505–508 (1985).

[b10] Wei L. F., Johansson J. R., Cen L. X., Ashhab S. & Nori F. Controllable coherent population transfers in superconducting qubits for quantum computing. Phys. Rev. Lett. 100, 113601 (2008). 18517785 10.1103/PhysRevLett.100.113601

[b11] Nalbach P. & Thorwart M. Landau-zener transitions in a dissipative environment: Numerically exact results. Phys. Rev. Lett. 103, 220401 (2009). 20366076 10.1103/PhysRevLett.103.220401

[b12] Shevchenko S., Ashhab S. & Nori F. Landau-Zener-Stückelberg interferometry. Phys. Rep. 492, 1 (2010).

[b13] Gasparinetti S., Solinas P. & Pekola J. P. Geometric landau-zener interferometry. Phys. Rev. Lett. 107, 207002 (2011). 22181761 10.1103/PhysRevLett.107.207002

[b14] Cheung D., Hoyer P. & Wiebe N. Improved error bounds for the adiabatic approximation. J. Phys. A: Math. Theor 44, 4153022 (2011).

[b15] Ferrón A., Domínguez D. & Sánchez M. J. Tailoring population inversion in landau-zener-stückelberg interferometry of flux qubits. Phys. Rev. Lett. 109, 237005 (2012). 23368247 10.1103/PhysRevLett.109.237005

[b16] Bason M. G. *et al.* High-fidelity quantum driving. Nat. Phys. 8, 147–152 (2012).

[b17] Wiebe N. & Babcock N. S. Improved error-scaling for adiabatic quantum evolutions. New J. Phys. 14, 013024 (2012).

[b18] Zhang Q., Gong J. B. & Wu B. Hierarchical theory of quantum adiabatic evolution. New J. Phys. 16, 123024 (2014).

[b19] Oliver W. D. *et al.* Mach-Zehnder interferometry in a strongly driven superconducting qubit. Science 310, 1653–1657 (2005). 16282527 10.1126/science.1119678

[b20] Sillanpää M., Lehtinen T., Paila A., Makhlin Y. & Hakonen P. Continuous-time monitoring of Landau-Zener interference in a cooper-pair box. Phys. Rev. Lett. 96, 187002 (2006). 16712390 10.1103/PhysRevLett.96.187002

[b21] Wilson C. M. *et al.* Coherence times of dressed states of a superconducting qubit under extreme driving. Phys. Rev. Lett. 98, 257003 (2007). 17678048 10.1103/PhysRevLett.98.257003

[b22] Izmalkov A. *et al.* Consistency of ground state and spectroscopic measurements on flux qubits. Phys. Rev. Lett. 101, 017003 (2008). 18764145 10.1103/PhysRevLett.101.017003

[b23] Rudner M. S. *et al.* Quantum phase tomography of a strongly driven qubit. Phys. Rev. Lett. 101, 190502 (2008). 19113251 10.1103/PhysRevLett.101.190502

[b24] Sun G. *et al.* Population inversion induced by LandauCZener transition in a strongly driven rf superconducting quantum interference device. Appl. Phys. Lett. 94, 102502 (2009).

[b25] LaHaye M. D., Suh J., Echternach P. M., Schwab K. C. & Roukes M. L. Nanomechanical measurements of a superconducting qubit. Nature 459, 960–964 (2009). 19536259 10.1038/nature08093

[b26] Sun G. *et al.* Landau-zener-stuckelberg interference of microwave-dressed states of a superconducting phase qubit. Phys. Rev. B 83, 180507 (2011).

[b27] Martinis J. M. Superconducting phase qubits. Quantum Inf. Process. 8, 81–103 (2009).

[b28] Simmonds R. W. *et al.* Coherent interactions between phase qubits, cavities, and TLS defects. Quantum Inf. Process. 8, 117–131 (2009).

[b29] Wen X. D., Zhu S.-L. & Yu Y. Dark periods in Rabi oscillations of a superconducting phase qubit coupled to a microscopic two-level system. Phys. Rev. B 80, 094507 (2009).

[b30] Sun G. *et al.* Quantum dynamics of a microwave driven superconducting phase qubit coupled to a two-level system. Phys. Rev. B 82, 132501 (2010).

[b31] Rubbmark J. R., Kash M. M., Littman M. G. & Kleppner D. Dynamical effects at avoided level crossings: A study of the landau-zener effect using rydberg atoms. Phys. Rev. A 23, 3107–3117 (1981).

[b32] Mullen K., Ben-Jacob E., Gefen Y. & Schuss Z. Time of zener tunneling. Phys. Rev. Lett. 62, 2543–2546 (1989). 10040015 10.1103/PhysRevLett.62.2543

[b33] Izmalkov A. *et al.* Observation of macroscopic landau-zener transitions in a superconducting device. Europhys. Lett. 65, 844–849 (2004).

[b34] Petta J. R., Lu H. & Gossard A. C. A coherent beam splitter for electronic spin states. Science 327, 669–672 (2010). 20133567 10.1126/science.1183628

[b35] Gaudreau L. *et al.* Coherent control of three-spin states in a triple quantum dot. Nat. Phys. 8, 54–58 (2011).

[b36] Cao G. *et al.* Ultrafast universal quantum control of a quantum-dot charge qubit using landau-zener-stückelberg interference. Nat. Commun. 4, 1401 (2013). 23360992 10.1038/ncomms2412 PMC3562462

[b37] Kayanuma Y. Stokes phase and geometrical phase in a driven two-level system. Phys. Rev. A 55, R2495–R2498 (1997).

